# Neon-green fluorescence in the desert gecko *Pachydactylus rangei* caused by iridophores

**DOI:** 10.1038/s41598-020-79706-z

**Published:** 2021-01-11

**Authors:** David Prötzel, Martin Heß, Martina Schwager, Frank Glaw, Mark D. Scherz

**Affiliations:** 1 Zoologische Staatssammlung München (ZSM-SNSB), Münchhausenstr. 21, 81247 Munich, Germany; 2 Department Biologie II, Ludwig-Maximilians-Universität München, Großhaderner Straße 2, 82152 Planegg-Martinsried, Germany; 3 Department of Applied Sciences and Mechatronics, Munich University of Applied Sciences, Lothstr. 34, 80335 Munich, Germany

**Keywords:** Herpetology, Biological fluorescence, Behavioural ecology

## Abstract

Biofluorescence is widespread in the natural world, but only recently discovered in terrestrial vertebrates. Here, we report on the discovery of iridophore-based, neon-green flourescence in the gecko *Pachydactylus rangei*, localised to the skin around the eyes and along the flanks. The maximum emission of the fluorescence is at a wavelength of 516 nm in the green spectrum (excitation maximum 465 nm, blue) with another, smaller peak at 430 nm. The fluorescent regions of the skin show large numbers of iridophores, which are lacking in the non-fluorescent parts. Two types of iridophores are recognized, fluorescent iridophores and basal, non-fluorescent iridophores, the latter of which might function as a mirror, amplifying the omnidirectional fluorescence. The strong intensity of the fluorescence (quantum yield of 12.5%) indicates this to be a highly effective mechanism, unique among tetrapods. Although the fluorescence is associated with iridophores, the spectra of emission and excitation as well as the small Stokes shifts argue against guanine crystals as its source, but rather a rigid pair of fluorophores. Further studies are necessary to identify their morphology and chemical structures. We hypothesise that this nocturnal gecko uses the neon-green fluorescence, excited by moonlight, for intraspecific signalling in its open desert habitat.

## Introduction

Biofluorescence in vertebrates is primarily known from marine organisms, such as reef fish^1,2^, catsharks^3^, and sea turtles^4^. However, especially over the last three years, a large number of terrestrial tetrapods have been discovered to fluoresce, including mammals^5,6^, birds^7–10^, amphibians^11–18^, and squamate reptiles^6,19–22^. This flurry of discoveries has fueled a major uptake in interest in this subject, in terms of phylogenetic, mechanistic, and functional diversity.

Although little is known about the function or evolution of biofluorescence^23,24^, numerous hypotheses have been put forward for different taxa, including photo-protection^25^, UV light detection^26^, prey^27^ or pollinator^28^ attraction, and signalling for mate choice, species recognition^2,3,10,29–32^, or male–male interactions^33^. Indeed, in some cases, fluorescence may simply be coincidental^12,34^.

As far as is known, all squamate reptiles and amphibians use only two fluorescence mechanisms: (1) the inherent fluorescence of bone^35^, or (2) chemicals circulated in the lymph. In amphibians, lymph-based fluorescence is thought to be widespread^15^, and has been well studied in treefrogs of the genus *Boana*^13,14^. However, bone-based fluorescence also occurs in a handful of species, such as the pumpkin toadlet genus *Brachycephalus*^11,36^. In squamates, all fluorescence known so far has been bone-based, made visible to the observer either by translucent skin, e.g. the geckos *Chondrodactylus bibronii*^20^, *Cyrtodactylus baluensis*^6^ and *Cyrtodactylus quadrivirgatus*^19^, or, in the case of chameleons, by specialised, transparent scales with dermal ‘windows’ that reside on outgrowths of the underlying bone^21^.

Here we report on the discovery of iridophore-based fluorescence, a novel mechanism of dermal fluorescence in the nocturnal web-footed gecko *Pachydactylus rangei* (Andersson, 1908). This well-known gecko lives in dunes and dry riverbeds in the Namib desert of Namibia^37^ and is unique among Gekkonidae due to its eponymous webbed hands and feet, which are an adaptation for effective sand shovelling^38^. We show that these geckos have bright neon-green fluorescent regions on the lower flank and around the eye and hypothesise a role for this phenomenon in visual signalling under moonlight.

## Results

**Figure 1:**
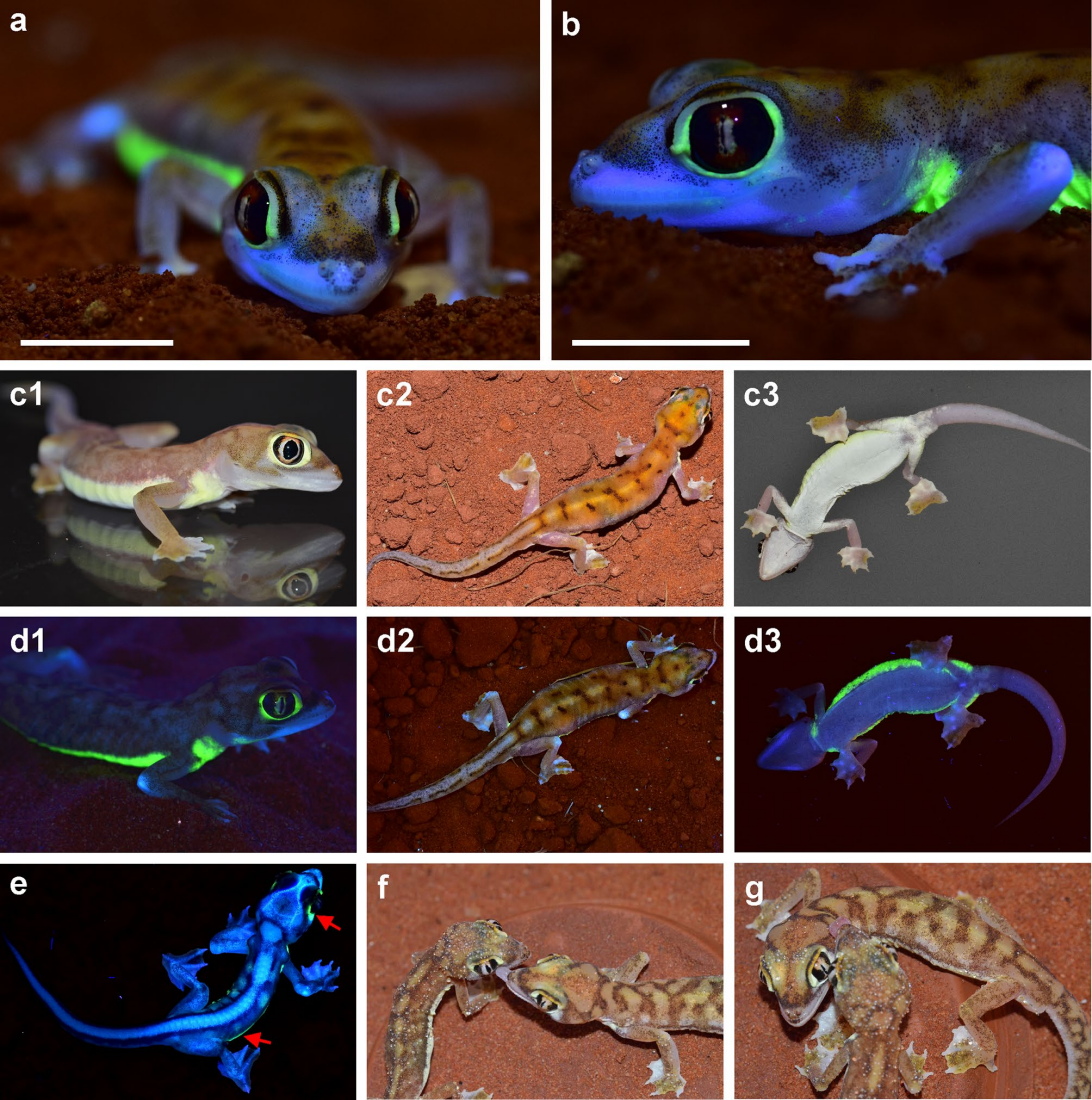
*Pachydactylus rangei* under UV illumination and under visible light. (**a**,**b**) Female *P. rangei* under UV illumination at 365 nm and (visible) dim background illumination; scale bars = 10 mm. (**c**) *P. rangei* under visible light in lateral (**c1**, male), dorsal (**c2**, female), and ventral view (**c3**, female). (**d**) The respective specimens illuminated with UV light (maximum emission at 365 nm) and dim background illumination in lateral view (**d1**, male), dorsal (**d2**, female) and ventral view (**d3**, female) showing neon-green fluorescence around the eyes and along a ventrolateral stripe. Note that the 365 nm light used to produce this fluorescence is far from the 465 nm peak excitation wavelength. (**e**) Hatchling, nine days post hatching, showing both the green dermal fluorescence seen in adults (indicated by red arrows) as well as blue bone fluorescence at the vertebrae, skull and the phalanges through its translucent skin. (**f**,**g**) *P. rangei* approaching each other and licking water from each other’s bodies (husbandry observation).

When illuminated with UV light *Pachydactylus rangei* shows a bright neon-green fluorescence in both sexes (Fig. 1, Supplementary Video 1), as well as in hatchling juveniles (Fig. 1e). The skin around the eyes and along the flank in a ventrolateral stripe becomes a strong neon-green colour when illuminated with light of about 365 nm (Fig. 1d). Under natural light conditions these parts of the skin are pale yellow, in contrast to the greyish white of some areas of the head and the sand-coloured dorsum (Fig. 1c,f,g). In specimens preserved in 70% ethanol, dermal fluorescence was only detected in specimens stored for a maximum of about 30 years. For example, out of 34 specimens preserved in 1988 (ZSM 307/1988/01-34), only six specimens still clearly fluoresced under UV light in 2020. The effect already begins to fade after a few years in preservative.

**Figure 2:**
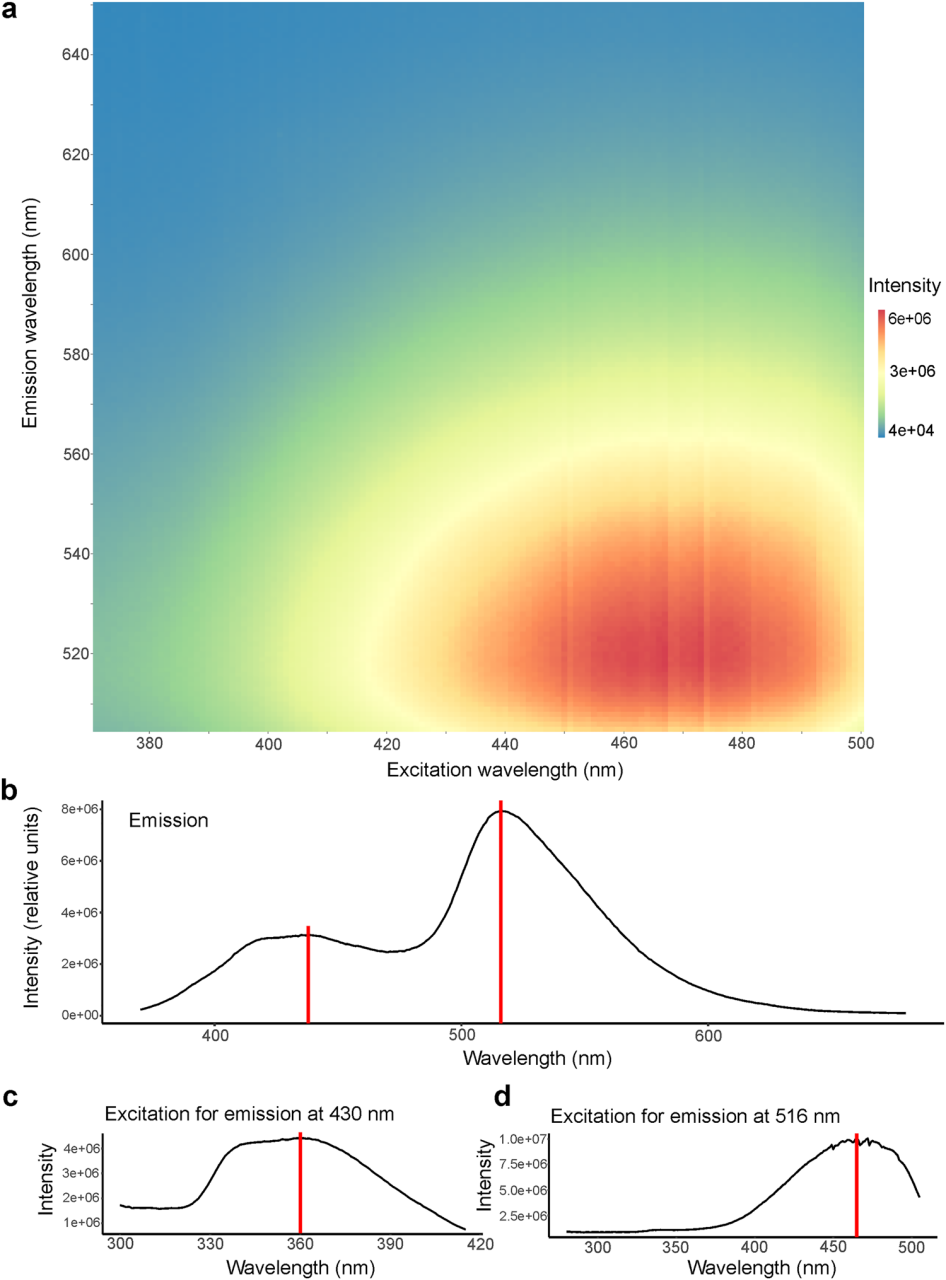
Excitation and emission spectra of fluorescence from the ventrolateral skin of *Pachydactylus rangei*. (**a**) Excitation-emission matrix (intensity in arbitrary units). (**b**) Emission spectrum with the maximum at 516 nm (neon-green) and smaller peak with a maximum around 430 nm (blue); both indicated by a red line. (**c**) Excitation spectrum for the smaller emission peak at 430 nm with the maximum at 360 nm (in the UV-A spectrum) indicated by a red line. (**d**) Excitation spectrum for the emission peak at 516 nm with the maximum at 465 nm (blue) indicated by a red line.

The emission maximum of this fluorescence is at a wavelength of 516 nm, in the greenish-yellow region of the visible spectrum (Fig. 2a,b). A second, lower peak is present around 430 nm in the blue region of the spectrum (Fig. 2b). The excitation maximum for this smaller emission peak at 430 nm is at 360 nm (in the UV-A spectrum; Fig. 2c) and for the emission maximum at 516 nm is at 465 nm (in the blue region of the spectrum; Fig. 2a,d). The quantum yield, Φ_f_, was determined to be 0.125 at 465 nm (12.5% of absorbed photons are re-emitted as fluorescence). In addition to the dermal fluorescence, blue, bone-based fluorescence is evident on the jaws, elbows, and digits under UV light due to the transparency of the skin, especially in juveniles (Fig. 1e).

**Figure 3:**
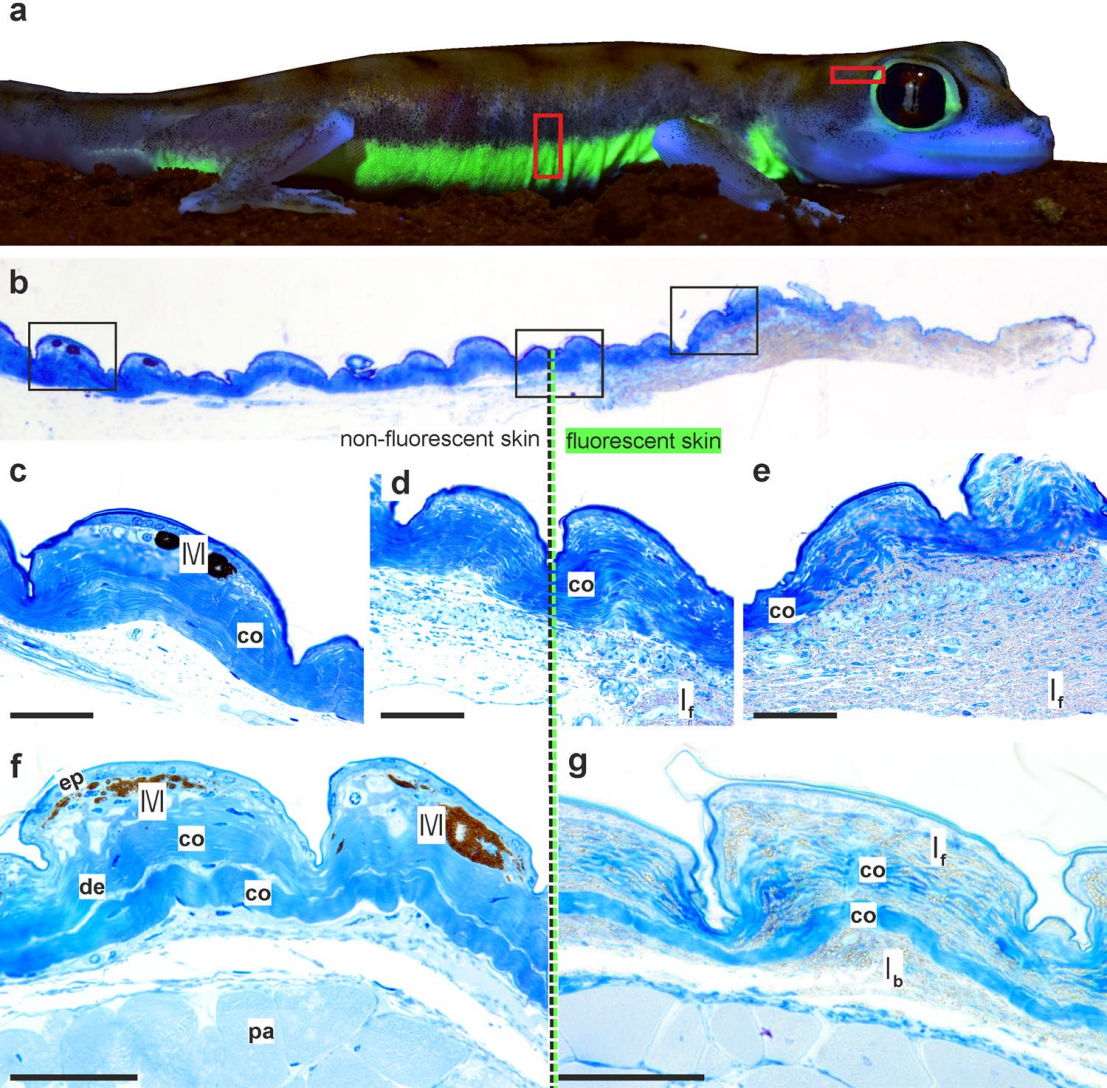
Histology of *Pachydactylus rangei* skin. (**a**) Boxes indicating schematic position of histological samples; (**b**) section from temporal region of eye with transition from non-fluorescent skin (left; posterior in a) to fluorescent skin (right, anterior in a); schematic boxes are enlarged below; (**c**) non-fluorescent skin containing melanophores; (**d**) transition between non-fluorescent to fluorescent skin; (**e**) fluorescent skin containing a large amount of iridophores with granular material; (**f**,**g**) sections from the ventrolateral stripe, non-fluorescent (**f**) and fluorescent (**g**). Scale bars = 50 µm. *co* collagen fibre, *de* dermis, *ep* epidermis, *I*_*f*_ fluorescent iridophores, *I*_*b*_ basal (non-fluorescent) iridophores, *M* melanophore, *pa* parenchymatous cells.

We examined histological sections of the skin of *P. rangei* to establish the localisation of its fluorescence. The skin of *P. rangei* has not previously been studied, but resembles in most respects the typical squamate structure (Fig. 3), composed of an outer layer of epidermis and an inner dermis^39,40^. The dermis contains layered collagen fibres, connective tissue cells and scattered chromatophores. Remarkably few chromatophores were observed in the investigated sections of skin compared to other gecko species^41,42^. Erythrophores or xanthophores were completely absent, and only small aggregations of melanophores were found centred in some of the tuberculate scales. This low density of pigment cells is unsurprising, given the rather translucent, pale skin of the gecko with isolated pigmented dots that can also be seen in macro-photographs (Figs. 1a,b, 3a).

**Figure 4:**
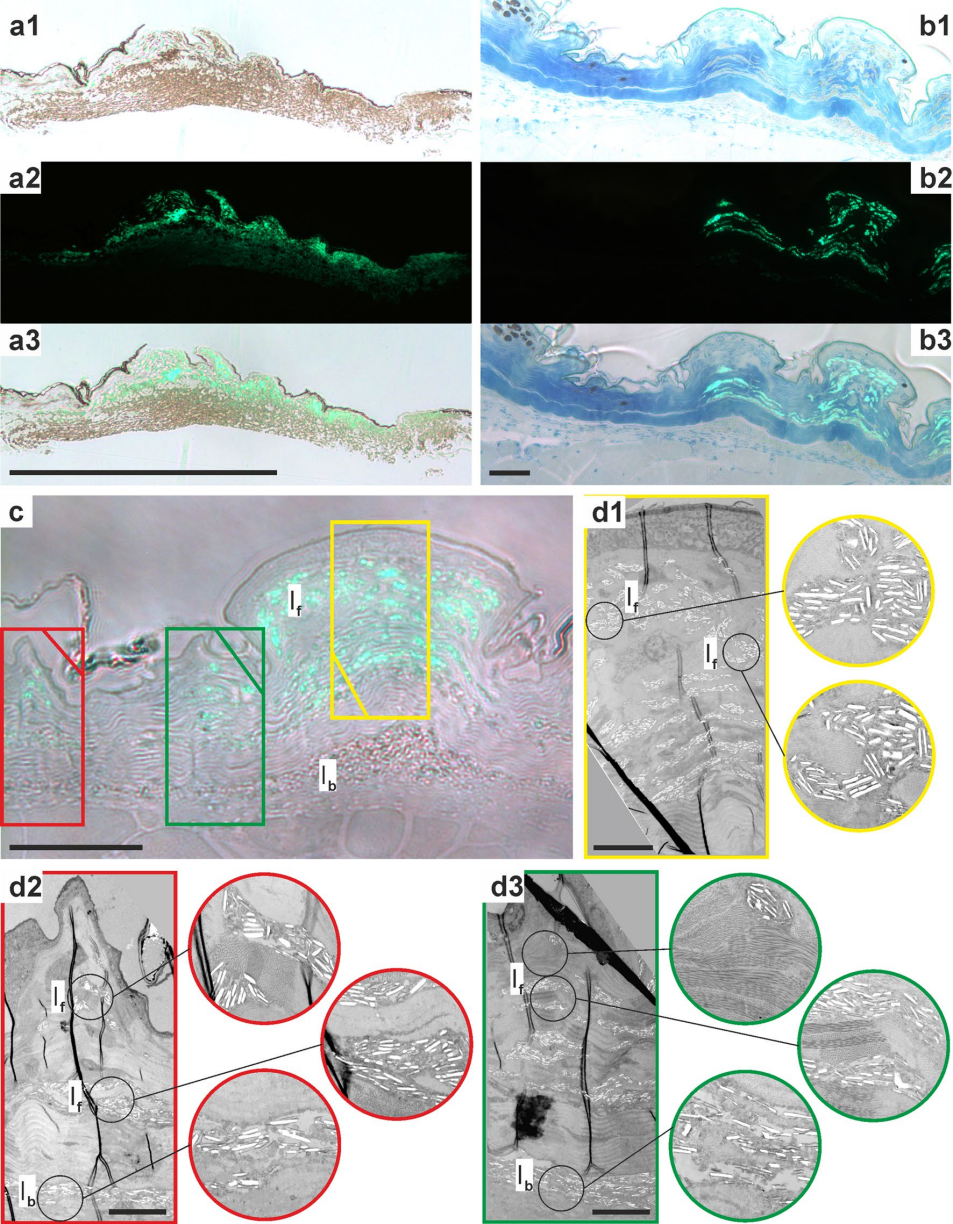
Distribution of iridophores in the dermis of *Pachydactylus rangei.* (**a**) Sections from skin around the eye; (**a1**) light microscopy (LM) image; (**a2**) fluorescence microscopy image; (**a3**) image overlay of a1 and a2 showing the fluorescence of the iridophores in the outer layers; scale bar: 500 µm. (**b**) LM sections from skin of ventrolateral stripe with the transition of non-fluorescent skin including melanophores (left) to fluorescent skin with iridophores (right); (**b1**–**b3**) following (**a1**–**a3**), scale bar: 50 µm; (**c**) section of fluorescent skin from the ventrolateral stripe in LM, scale bar: 50 µm; (**d**) Transmission electron microscopy (TEM) images of the sectors indicated (by colour) in (**c**), with (**d1**) in yellow, (**d2**) in red, and (**d3**) in green; circled images show enlarged iridophores of the respective image top down, scale bar: 10 µm. Note how the non-fluorescent iridophores of the lowest layer are disordered and more loosely packed than the fluorescent iridophores above. *I*_*f*_ fluorescent iridophores, *I*_*b*_ basal (non-fluorescent) iridophores.

By taking sections at the transition between fluorescent and non-fluorescent regions on the body (behind the eye and on the flank), we were able to directly compare these regions to identify the fluorescent structures. In fluorescent regions, large numbers of granules, considered to be guanine crystals^41,43^, were concentrated in irregularly shaped iridophores amongst the regular collagen matrix of the skin (Fig. 3b,e,g). In the non-fluorescent parts, iridophores and their tell-tale guanine crystals were completely absent, but a few melanophores were present in the elevated scales (Fig. 3b,c,f). Using a fluorescent microscope with excitation of blue light (450–490 nm), we established that it is the iridophores in fluorescent regions of skin that produce green fluorescence (500–550 nm), but that the innermost iridophores lying below the collagen layers do not fluoresce (Fig. 4).

Transmission electron microscopy (TEM) revealed the guanine crystals to be consistent with those observed in other gecko skin (e.g.^41,42,44^), though less organised than in some other geckos^42^. We found that the iridophores in the non-fluorescent regions did not differ strongly from those from the fluorescent regions, but do have marginally less regularly angled (two-tailed Wilcoxon Rank Sum test, W = 24,925, *p* = 0.03189; Supplementary Figure 1a) and slightly longer (W = 23,382, *p* = 0.001719; Supplementary Figure 1b) guanine crystals. These guanine crystals from non-fluorescent regions also appear to be more loosely packed than those in fluorescent regions. We consider these to represent two separate classes of iridophores, which we term ‘basal’ iridophores and ‘fluorescent’ iridophores (Fig. 4d). These iridophore classes are distinguished primarily on the basis of their localisation (basal versus superficial, respectively) and fluorescence (non-fluorescent versus fluorescent, respectively).

## Discussion

### A novel fluorescent mechanism

The neon-green fluorescence of *P. rangei* is the first documented case of dermal fluorescence in a squamate reptile. The fluorescence is remarkably bright, with a quantum yield of 12.5%, and is evidently associated with a special fluorescent class of iridophores in the dermis. These fluorescent iridophores differ from basal iridophores, which do not fluoresce, by having more ordered, slightly shorter, and more tightly spaced guanine crystals (Figs. 3, 4, S1).

Iridophores have a well-known role in squamate skin colouration and physiologically controlled colour change^41–43,45^. Until now, squamate iridophores have only been known to reflect light, with colour achieved by interference effects^42,43^. In several species of reef fish, however, guanine crystals in the eye ring and parts of the head or thorax have been found to exhibit red fluorescence under blue light, with a maximum emission at ca. 600 nm^32^. In addition to typical red-fluorescent guanine platelets, one guanine crystal fluoresced blue-green^2^. This might be interpreted as evidence that the guanine crystals themselves may be the fluorescent materials in the skin of *P. rangei*. However, guanine’s standard fluorescence has an excitation maximum between 170 and 290 nm, and emission maximum near 350 nm^46^. It thus has a sizeable Stokes shift of 60–180 nm and fluoresces in the near-UV. By contrast, the fluorescence of *P. rangei* exhibits two peaks, a lower one with an excitation maximum at 360 nm and emission maximum at 430 nm (Stokes shift 70 nm), and a higher one with an excitation maximum at 465 nm and emission maximum at 516 (Stokes shift ca. 50 nm). These small Stokes shifts argue for rigidity of the chromophores’ structures in their electronic ground and excited states^47^. The similarity of the equilibrium structures in the electronic ground and excited states promotes a high quantum yield due to a large transition moment. The low energy loss due to vibrational relaxation is typical for compounds with an extended aromatic π-electron system, which promotes the suppression of nonradiative relaxation due to the rigidity of the structure^48^. Since the first fluorescence maximum is located in the excitation region for the second emission peak, the emitted light can be reabsorbed from the second chromophore for excitation thus leading to an enhanced emission in the greenish region at around 516 nm and a decrease in the emission at 430 nm. By this mechanism the skin can use not only blue but also light of the near-UV for a strong emission in the green region.

Although iridophores of geckos have been rather poorly studied, it is clear that they are diverse in structure and the shape of the guanine crystals they contain^40^. In the tokay (*Gekko gecko*), iridophores are the most superficial layer of chromatophores in blue-coloured areas of the skin, but lie beneath erythrophores in red areas^41^. In the diurnal *Phelsuma* geckos, iridophores reflect light through xanthophores or erythrophores, producing different colours by interference effects: the guanine crystals are ordered in green or blue skin and disordered in white or red skin^42^. The leopard gecko (*Eublepharis macularius*), on the other hand, lacks iridophores altogether^49^. Until now, however, it has been thought that all geckos have a single class of iridophore^43^, making *P. rangei* the first known gecko to have two distinct iridophore classes.

Chameleons are also known to have two different types of iridophores, with superficial (S-) iridophores with small, tightly packed guanine crystals, that are implemented in their colour change, and deep (D-) iridophores containing larger, brick-shaped and disorganized guanine crystals that reflect in the near-infrared region (700–1400 nm), presumably supporting thermoregulation^43^. *P. rangei* does not have the ability to change its colour substantially, and its fluorescent iridophores do not resemble the superficial iridophores of chameleons. Although the basal iridophores of *P. rangei* also do not closely resemble those of chameleons, we suspect that they serve a similar purpose, namely acting as an internal ‘mirror’. This ‘mirror’ function serves both to reflect infrared radiation as hypothesised in chameleons^43^ and also possibly to amplify non-directionally emitted fluorescence. Establishing how the two classes of iridophores differ in gene expression and protein complement, and when in embryonic development this differentiation occurs (given that freshly hatched geckos already show the fluorescent patterns; Fig. 1e) will be important in deepening our understanding of the evolutionary origin of this novel trait.

### Biological significance

Biological relevance is the most prominent issue facing all studies revealing biofluorescence in organisms^6,34^. So far, only few studies have managed to provide clear evidence of biological significance^9^. In some taxa a strong case can be made: In chameleons, for example, special raised outgrowths of the bone lead to fluorescence in areas that are associated with sexual selection^21^. Another example is the frog *Boana punctata*, where fluorescence is due to fluorescent Hyloin-derivatives produced by lymph and glandular tissues^13^. The fluorescence enhances the brightness of the frog under low light conditions by converting irradiance from the UV-spectrum to longer wavelength emission, where their visual sensitivity is higher^12,13^. In other cases, we suggest that fluorescence is purely coincidental, especially those where bones, which are inherently fluorescent, have been found to fluoresce visibly through non-specialized skin under strong UV light^6,19,20^. We contend that the fluorescence of *P. rangei* has strong potential to be biologically significant on three grounds: (1) brightness, (2) placement, and (3) behavioural and ecological indications.

Although many observations of fluorescence in nature have been published, calculations on quantum yields of the respective fluorophores are still rare. Yet, quantitative studies are needed in order to evaluate the biological significance of fluorescence^34^. With a quantum yield of 12.5% on the skin (not on an extracted fluorophore), the fluorescence of *P. rangei* is among the strongest known in vertebrates, being on the level of that achieved by the tree frog *B. punctata* (12 ± 3%)^13^. These two species are brighter than other animals measured in vivo, such as the 4.2% of fluorescent feathers in the parrotlet *Forpus xanthopterygius*^10^ or the 0.29% of the bone-based fluorescence in chameleons^21^.

The fluorescent areas of *P. rangei* are concentrated around the eyes and along the lower flanks. This positioning is practically invisible to predators with a higher perspective (e.g. birds and jackals; Fig. 1d2), but highly conspicuous from a gecko’s perspective (Fig. 1d1). As *P. rangei* is sociable but generally solitary, and occurs at low population densities, such a signal might serve to locate conspecifics over greater distances, in lieu of the conspicuous auditory signals employed by some other geckos for long-distance communication^50^. Encounters in *P. rangei* might serve purposes beyond mating opportunities: as the Namib desert has extremely low precipitation, fog is a key water source for its flora and fauna^51,52^. Fog condenses on the bodies of the geckos, and they lick it from their faces. In husbandry, we have observed individuals licking water from conspecifics (Fig. 1f,g)^53^, taking advantage of a much greater available surface area. Additionally, after short periods of isolation, the geckos run to meet each other (DP, personal observation). The combination of vital hydration with socialisation might reinforce signals that enable such meetings, and the cost of visibility to predators with higher vantage points, might constrain the signals to regions best visible from eye-level and below.

*Pachydactylus rangei* is a strictly nocturnal gecko, with activity beginning between 20:30^37^ and 23:00 (Schmidt, personal communication). Any fluorescence it achieves must therefore be from ambient light, provided almost exclusively by moonlight. In the Namib, moonlight can be very bright during the full-moon half of the moon phase, and some nocturnal spiders are most active when the moon phase is dimming in order to avoid visual predators^54^. Given the clearer skies and lack of canopy compared to the habitat of *Boana punctata*, the fluorescence of *P. rangei* is more likely to be visible in situ, and thus biologically relevant. Moreover, nocturnal geckos often have extremely good low-light colour vision due to their three cone types and absence of rods on the retina^55,56^. The vision of *P. rangei* remains to be studied in detail but is likely to be likewise highly acute in darkness.

### Outlook

In summary, *P. rangei* exhibits some of the brightest fluorescence among tetrapods. The fluorescence is localised to one of two specific classes of iridophores in the dermis. Although fluorescence is associated with iridophores, the spectra and the Stokes shift argue against guanine crystals as its source, but rather a rigid pair of fluorophores. Such an arrangement might, for example, be found in a fluorescent-iridophore-specific protein. The precise molecular basis of this fluorescence must still be established, and further studies using comparative proteomic and transcriptomic approaches will help to elucidate the chemical(s) responsible for this bright fluorescence. Of particular interest will be the developmental patterning of fluorescence, which is completed during embryonic development inside the egg. For these methods, a whole genome sequence would also be a useful resource.

Based on the brightness, localisation and ecology of these geckos we consider it likely, that fluorescence plays a role in their social interactions. A key future step will be to carry out behavioural trials to establish whether fluorescence plays a role in territoriality or mate selection. Answering these questions will yield valuable insights into understanding one of the most remarkable cases of vertebrate fluorescence.

## Methods

### Specimens

55 specimens of different age and sex of *P. rangei* from the collection of the Zoologische Staatssammlung München, Germany (ZSM-SNSB) were tested for fluorescence under a UV LED torch (see below). Additionally, several live specimens from the private husbandry of DP were observed and photographed between 2017 and 2020. One captive bred female (ZSM 5/2020) died of natural causes in 2019 and was immediately stored in a freezer at − 20 °C. This fresh specimen was used for spectroscopy. Another captive bred specimen (uncatalogued) that died in 2020 of natural causes was preserved in aldehyde fixative for histological investigations shortly after its death.

### Ethics

No experiments were undertaken on live animals, and this observational study does not fall under German Animal Experimentation Law (Tierschutzgesetz §§7–10a). Photographs are of captive-bred animals kept for husbandry purposes. Photographs were taken in a non-invasive manner, without disturbing the animals.

### Fluorescent photography

Photographs were taken with Nikon SLR cameras (D5100 and D7300), using a Tamron SP AF 90 mm f/2.8 Di macro lens. Under the dim light conditions emitted by a standard LED ceiling light the specimen was additionally illuminated by UV light produced by a Tank007-TK-566 flashlight (3 W) or a TATTU U3S UV flashlight, both emitting at a maximum of 365 nm at a distance of about 20 cm from the specimen.

### Fluorescence spectroscopy

Fluorescence excitation and emission spectra were recorded with a Fluorolog (Horiba) fluorimeter. All fluorescence spectra were corrected for the wavelength-dependent output of the Xenon lamp. Intensity of fluorescence is given by the detected emission divided by the simultaneously measured intensity of the lamp at the same wavelength. The monochromatic beam irradiated an area of 1 × 9 mm of the sample. The sensor was focused on a 20 × 10 mm sized part of the skin, which was dissected from the fluorescent ventrolateral stripe of the *P. rangei* specimen ZSM 5/2020*.* Recorded spectra were as follows:

1. *Emission spectrum.* The light emitted by the fluorescence from 370 to 680 nm (max. fluorescence emission at 516 nm) was recorded at an excitation wavelength of 350 nm (in order to avoid overlapping of emission and excitation wavelengths), with an increment of 1.00 nm and a bandpass of 1.70 nm.
2. *Excitation spectrum for the emission at 516 nm (*= *max. emission wavelength).* The excitation wavelengths from 280 to 505 nm were recorded with an increment of 1.00 nm and a bandpass of 1.30 nm.
3. *Excitation spectrum for the emission at 430 nm (*= *second peak of max. emission wavelength).* The excitation wavelengths from 300 to 415 nm were recorded with an increment of 1.00 nm and a bandpass of 2.20 nm.
4. *Excitation-emission matrix*. The excitation wavelengths from 370–500 nm, and the emission wavelengths from 505 to 650 nm were recorded; both with an increment of 1.00 nm and a bandpass of 1.30 nm. Note: The maximum emission could not be monitored over its full width in the excitation-emission matrix due to the small Stokes shift (ca 50 nm), which means the emission and excitation maxima are close together and monitoring could only be started 12 nm below the emission spectrum in order to avoid detection of the exciting radiation in the emission spectrum.

### Quantum yield calculation

The quantum yield of the fluorescent ventrolateral stripe of the *P. rangei* specimen (ZSM 5/2020) was calculated, following largely Iriel and Lagorio^57^ and Taboada et al.^13^. The collection of photons was performed under a fixed sample-detector geometry so that the solid angle of the detected radiation was constant for all measurements. All spectra were recorded with the same bandpass (1.3 nm). Using the Fluorolog fluorimeter (see above) the following five measures were determined:

1. *Emission spectrum from the fluorescent skin*. The light emitted by the fluorescence from 478 to 680 nm (max. fluorescence emission at 516 nm) was recorded under excitation at 466 nm; the area under this emission curve represents the number of photons emitted by the sample as fluorescence (J_f_) in the detected spatial segment.
2. *Integrated reflected light from the blank*. The light scattered from a blank (barium sulfate, BaSO_4_) was collected from 456 to 476 nm using the same excitation wavelength (466 nm). The spectrum was recorded using a UV fused silica metallic filter (Newport FSQ-ND3.0) to avoid detector damage. The integrated area under this curve is denoted with J_0_.
3. *Integrated reflected light from the fluorescent skin.* The same procedure as (2) but using fluorescent skin instead of a blank. The area under the curve is the light reflected by the skin (J).
4. *Scattered light intensity from the fluorescent skin.* The excitation light was fixed at 700 nm (a wavelength where the fluorescent skin essentially did not absorb light). The emerging light from the sample was recorded as a function of wavelength between 690 and 710 nm with the same filter as used in (2). The area under the curve (I) represents the scattered light intensity from the sample.
5. *Scattered light intensity of the blank.* Integrated scattered light intensity for the blank (I_0_). The spectrum was recorded as in step (4) using barium sulphate.

The fluorescence quantum yield (Φ_f_) is defined as the fraction of photons of the absorbed incident radiation. It is emitted as fluorescence light and was calculated as follows:

$${\Phi }_{f}=\frac{{\mathrm{number}\,\mathrm{of}\,\mathrm{emitted}\,\mathrm{photons}}}{{\mathrm{number}\,\mathrm{of}\,\mathrm{absorbed}\,\mathrm{photons}}}=\frac{{J}_{f}}{{J}_{0} \left(\frac{I}{{I}_{0} R}\right) -J}$$

In the above equation differences in scattering characteristics between the total reflecting BaSO_4_ blank and the specimen are taken into account. With the correction factor I/I_0_ differences in surface texture of the skin and the blank are considered. Since the surface quality can lead to varying angular dependent spatial photon distributions of the scattered light it is necessary to collect photons in the same spatial angle by irradiating with the same monochromatic light. The ratio of both integrated signals reflects differing reflection features of skin and reference sample. Furthermore, the skin is not totally transmissive, but is also absorbing a part of the incident light. This characteristic is considered by the relative reflectance, R, which is the ratio of the integrated angular dependent amount of reflected light from the sample and the integrated angular dependent amount of the reflected light of the reference plate (BaSO_4_). With an incident radiation of 700 nm this ratio R amounts to 0.84 with an error of approximately 10% due to variation in the reflectance by irradiating different sites of the skin (see Supplementary Fig. S2 and Supplementary Table S1 for additional information).

### Histology

Skin from the anterior eye region and of the ventrolateral flanks of the *P. rangei* specimen (ZSM 5/2020) was dissected with a razorblade, in both cases taking strips of skin with adjoining non-fluorescent skin. Samples were stored in 2.5% glutaraldehyde in 0.1 M phosphate buffer.

The excised skin was washed in 0.1 M phosphate buffer, stained in buffered 1% Osmium tetroxide (OsO_4_) for 1 h on ice, washed again, dehydrated in a graded acetone series, embedded in epoxy resin^58^, sectioned at 1 µm using a RMC MT-7000 ultramicrotome with a Diatome Histo Jumbo diamond knife, and mounted on glass slides. Some slices were stained with a 1:1 mixture of methylene blue and Azure II for ca. 5 s at 80 °C^59^. Stained slices were sealed under coverslips with DPX mounting medium (Agar Scientifics). The glass slides were imaged in bright field illumination using an Olympus CX 41 light microscope with a DP25 digital camera (objectives: Olympus Plan C 10 × NA 0.25, Olympus UPlanSApo 40 × NA 0.95).

### Fluorescence microscopy

Stained and unstained slices were photographed with a Leica DM 750 fluorescence microscope, equipped with Leica DMC 4500 digital camera, a CoolLED *p*E-300 white light-source using the GFP filter cube (excitation wavelength at 475 nm; objectives Leica HI Plan 10 × NA 0.25 and 40 × NA 0.65) or in transmission bright field.

### Transmission electron microscopy

Subsequent to the semithin section series some ultrathin sections (70 nm) were cut with a diamond knife using a Leica EM UC6 ultramicrotome, and mounted on formvar-coated copper slot-grids (Agar Scientific G2500C). The slices were double-stained with 8% uranyl acetate and lead citrate^60^ and imaged with a FEI Morgagni 268 TEM at 80 kV.

### Guanine crystal length and angle

The length and angle of guanine (or guanine-like) crystals in TEM images were measured in ImageJ2^61^ using the straight line tool based on reoriented slices. Differences between fluorescent and non-fluorescent iridophore guanine crystals were assessed based on two-tailed Wilcoxon Rank Sum tests in R 3.6.3^62^ and visualised using ggplot2^63^.

## Supplementary Information

Supplementary Infomations.

Supplementary Video 1.
