# Supplementary material for: Neon-green fluorescence in the desert gecko Pachydactylus rangei caused by iridophores
Source: Sci Rep. 2021 Jan 11;11:297. doi: 10.1038/s41598-020-79706-z (PMC7801506; doi:10.1038/s41598-020-79706-z)
Supplement: Supplementary file 1 — Supplementary Infomations. [file 41598_2020_79706_MOESM1_ESM.pdf]

Supplementary Information for

# Neon-green fluorescence in the desert gecko *Pachydactylus rangei* caused by iridophores

David Prötzel[1,*], Martin Heß[2], Martina Schwager[3], Frank Glaw[1] & Mark D. Scherz[1]

[1]Zoologische Staatssammlung München (ZSM-SNSB), Münchhausenstr. 21, 81247 München, Germany

[2]Department Biologie II, Ludwig-Maximilians-Universität München, Großhaderner Straße 2, 82152, Planegg-Martinsried, Germany

[3]Department of Applied Sciences and Mechatronics, Munich University of Applied Sciences, Lothstr. 34, 80335, München, Germany

[*]Corresponding author, e-mail: david.proetzel@mail.de

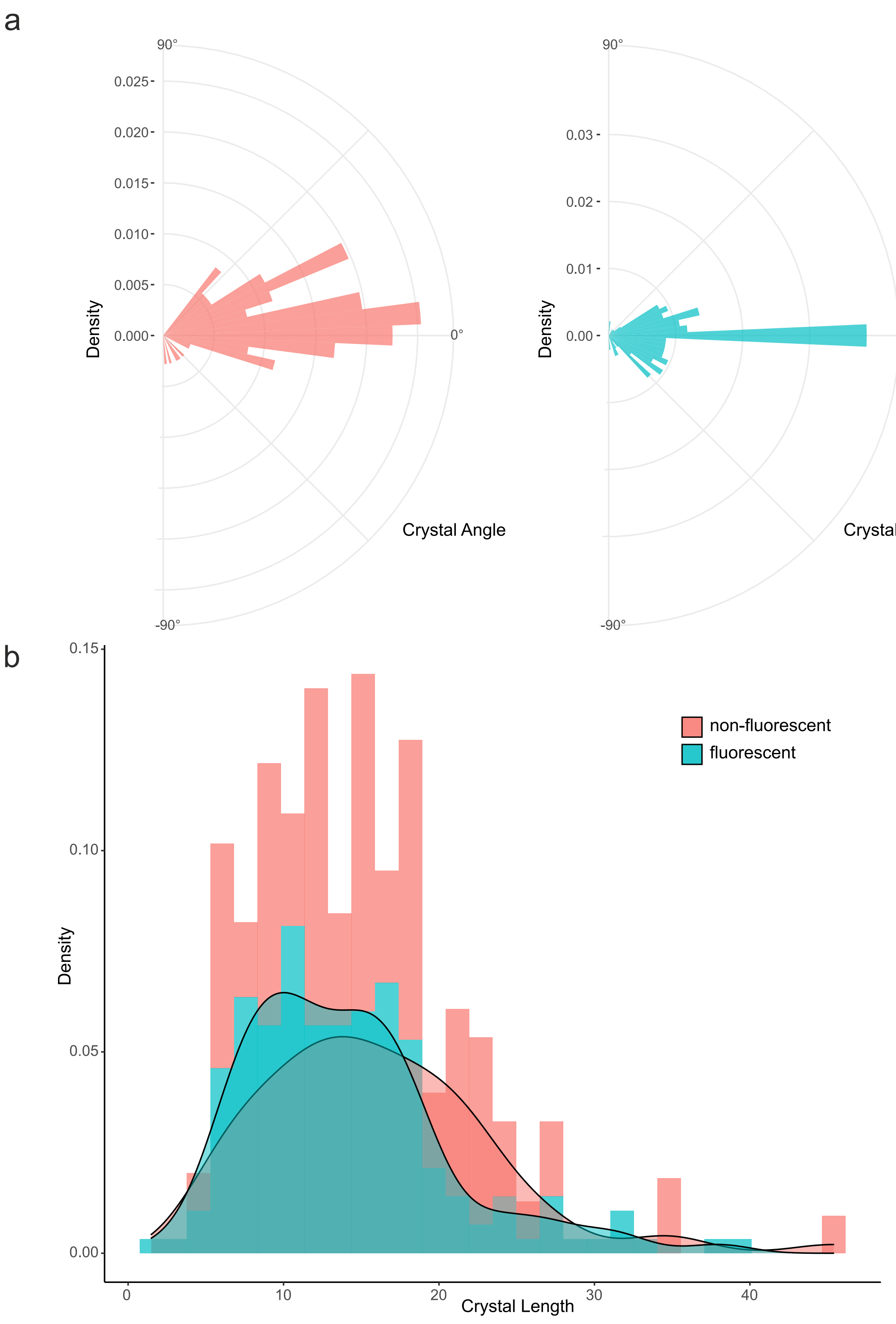


**Supplementary Figure S1.** (**a**) Crystal angle relative to a consensus angle of the skin in fluorescent (green) and non-fluorescent iridophores (red). (**b**) Histogram and density plot of crystal length in fluorescent and non-fluorescent iridophores.

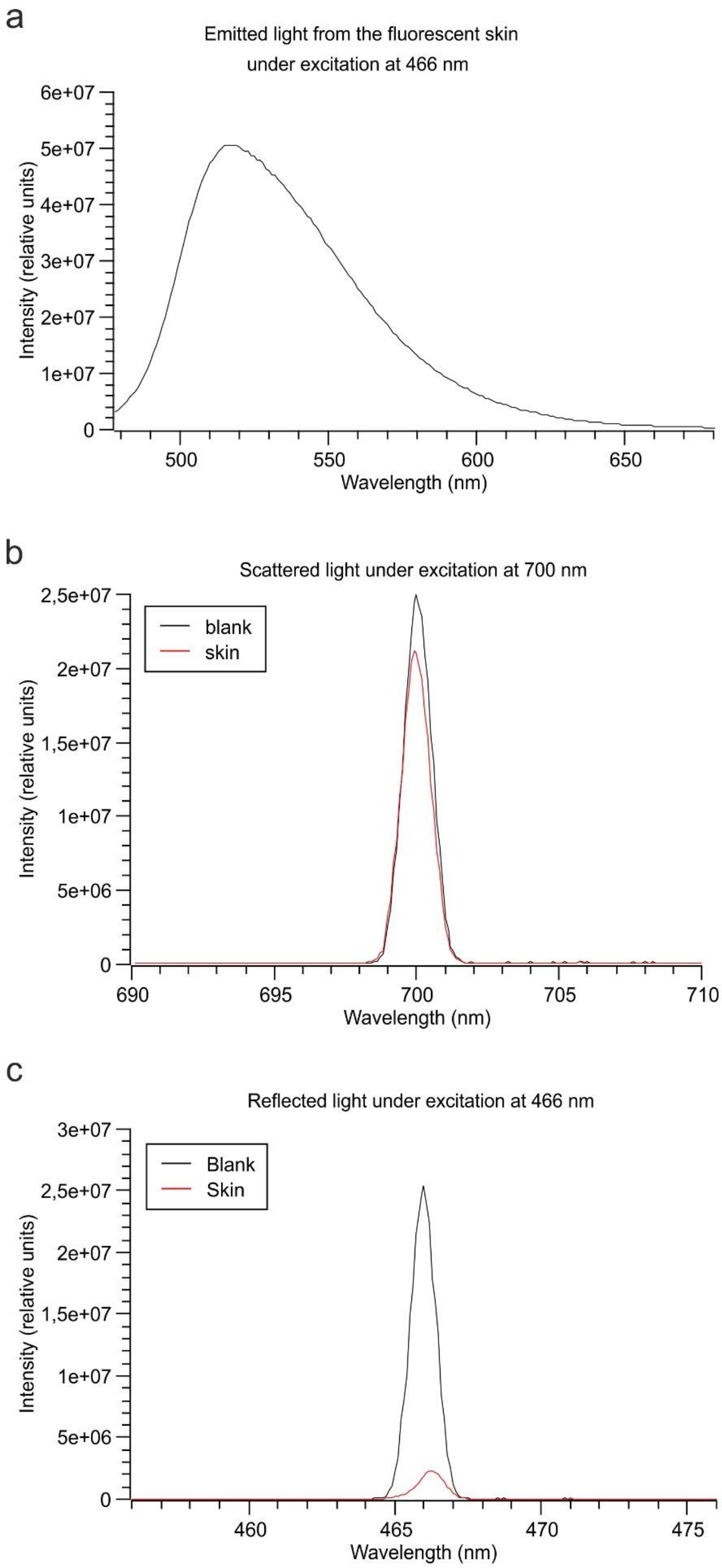


**Supplementary Figure S2.** Graphs illustrating the quantum yield calculation supplementing the respective section in 'Methods'. **(a)** Integrated area under the emission spectrum is denoted with $J_f$. **(b)** Integration of the blank and skin spectra yields $J_0$ and J respectively. **(c)** Integration of the blank and skin spectra yields $I_0$ and I respectively.

**Supplementary Table S1.** Integrated areas under the curves of Supplementary Fig. S2 used for the quantum yield calculation. Definitions of the integrated areas can be found in the Methods.

| **Integrated areas:** | **J** | **$J_0$** | **$J_f$** | **I** | **$I_0$** |
|---|---|---|---|---|---|
| **Calculated number of photons:** | $3.32 \times 10^9$ | $2.83 \times 10^{10}$ | $3.12 \times 10^9$ | $2.40 \times 10^{10}$ | $2.81 \times 10^{10}$ |
